# Functionalization of an extended-gate field-effect transistor (EGFET) for bacteria detection

**DOI:** 10.1038/s41598-022-08272-3

**Published:** 2022-03-15

**Authors:** Lea Könemund, Laurie Neumann, Felix Hirschberg, Rebekka Biedendieck, Dieter Jahn, Hans-Hermann Johannes, Wolfgang Kowalsky

**Affiliations:** 1 Institut für Hochfrequenztechnik, Technische Universität Braunschweig, 38106 Braunschweig, Germany; 2 Institute of Microbiology and Braunschweig Integrated Centre of Systems Biology (BRICS), Technische Universität Braunschweig, 38106 Braunschweig, Germany; 3 Cluster of Excellence PhoenixD (Photonics, Optics, and Engineering—Innovation Across Disciplines), 30167 Hannover, Germany

**Keywords:** Engineering, Electrical and electronic engineering, Biological techniques, Lab-on-a-chip

## Abstract

Traditional sensing technologies have drawbacks as they are time-consuming, cost-intensive, and do not attain the required accuracy and reproducibility. Therefore, new methods of measurements are necessary to improve the detection of bacteria. Well-established electrical measurement methods can connect high sensitive sensing systems with biological requirements. One approach is to functionalize an extended-gate field-effect transistor’s (EGFET) sensing area with modified porphyrins containing two different linkers. One linker connects the electrode surface with the porphyrin. The other linker bonds bacteria on the functional layer through a specific peptide chain. The negative charge on the surface of the cells regulates the surface potential which has an impact on the electrical behavior of the EGFET. The attendance of attached bacteria on the functionalized sensing area could successfully be detected.

## Introduction

Bacteria are often seen as pathogenic organisms but essential functions, for instance in healthcare and food safety, cannot be solved without microorganisms^1,2^. Nevertheless, the few pathogenic microorganisms can threaten human’s health dramatically^2^. *Escherichia coli* *(E. coli)* or *Staphylococcus aureus* are causing, inter alia, diarrhea, anemia, or kidney failure. Many infections can be treated with suitable antibiotics. But, as a consequence, an increase of resistant germs is observed^3^. Therefore, it is essential to detect pathogenic organisms at an early stage. Common detection methods can be enzyme-linked immunosorbent assay (ELISA) or polymerase chain reaction (PCR). ELISA profits from a fast response but errors in the detection process are possible^4^. PCR has a high sensitivity and accuracy but requires a large quantity of materials, educated personnel, and time^5^. In contrast, biosensors based on bioelectronics have a fast response, improve the sensitivity, and even reduce the sample volume drastically compared to traditional sensing technologies^6,7^. Biosensors can be used at doctor’s practice and enable a fast diagnostic with a subsequent therapy. Fatal infections as tuberculosis or HIV can be drastically reduced in developing countries^8^. In the last years, the amount of published extended-gate or floating-gate field-effect transistors (FET) as biosensors has seen an increase^6,9–13^. For instance, White et al.^10^ published a side-gated FET to detect hybridized desoxyribonucleic acid (DNA). Sheibani et al.^9^ documented an extended-gate FET (EGFET) to detect cortisol in human sweat.

Our work focuses on a label-free detection of whole *E. coli* K12 cells without an extensive sample preparation. The sensing area of an EGFET is functionalized by modified porphyrins and further covered with a liquid containing bacteria. The negative charge on the surface of bonded *E. coli* cells might have an impact on the electrode’s surface potential. A change is reflected in the electrical characteristic of the EGFET.

Previous publications modified electrode surfaces with different bioreceptors as antibodies, aptamers, or bacteriophages^14–16^. As alternative, bio-inspired porphyrins are of great interest due to their biological compatibility, their fluorescence in the visible range, their chemical stability under ambient condition, and their simple chemical accessibility^17^. So far, sensor systems have been functionalized by porphyrins to detect for example magnesium (II) ions, histidine, or DNA^18–20^.

Our developed EGFET as biosensor for the detection of bonded bacteria is introduced. Two operation modes of the EGFET are presented to detect the attendance of bacteria. Essential comparisons between measurement results recorded with and without bacteria are shown to clearly relate the electrical response to the attendance of *E. coli*. Finally, it is discussed that the functionalization is necessary for a significant signal.

## Experimental methods and materials

### Materials

Borosilicate glass were purchased from SCHOTT AG (Mainz, Germany). Solvents were purchased from SIGMA-ALDRICH® a brand of Merck KGaA (Darmstadt, Germany). Photoresist, thinner, and developer for lithography were purchased from MicroChemicals GmbH (Ulm, Germany). The negative photoresist AZ® nLof2035 were diluted with the thinner AZ® EBR Solvent with a mass ratio of 2:1. In general, deionized water was used. Phosphate-buffered saline (PBS) were purchased from Carl Roth GmbH + Co. KG (Karlsruhe, Germany) as tablets. It contains 0.14 mol/L NaCl, 2.7 mmol/L KCl, and 10 mmol/L phosphate with a pH-value of 7.4 ± 0.05 dissolved in deionized water. The ingredients of the lysogeny-broth medium (trypton, yeast extract, sodium chloride) were also purchased from Carl Roth GmbH + Co. KG (Karlsruhe, Germany).

### Sample preparation

The electrodes of the sensing area were realized on 0.7 mm thick borosilicate glass with a lift-off procedure. Glass substrates were cut into quadratic pieces with an edge size of 76 mm and were cleaned with acetone and isopropanol in an ultrasonic bath. Afterwards, the substrates were spin coated with the diluted negative photoresist AZ® nLOF2035 with a final speed of 3,000 rpm for 20 s. The samples were then heated on 70 °C to prevent small flaws and were further soft baked at 100 °C for 1 min. The samples were exposed by a high-pressure mercury lamp on a MA8/ BA6 System from SÜSS MicroTec SE (Garching, Germany). The exposed radiation energy was 100 mJ/cm^2^. Lithography mask and sample were in hard contact mode. The post exposure bake was performed as the soft bake. Photoresist in not exposed areas were removed with the developer AZ® 826 MIF for 1.5 min and cleaned with deionized water afterwards.

After metallization with 3 nm chromium and then 100 nm gold by electron beam evaporation, the lift-off process was performed. The metallized photoresist was removed by dimethyl sulfoxide (DMSO) under the influence of ultrasonic for 30–60 min.

### Formation of self-assembled monolayers (SAM)

**Figure 1:**
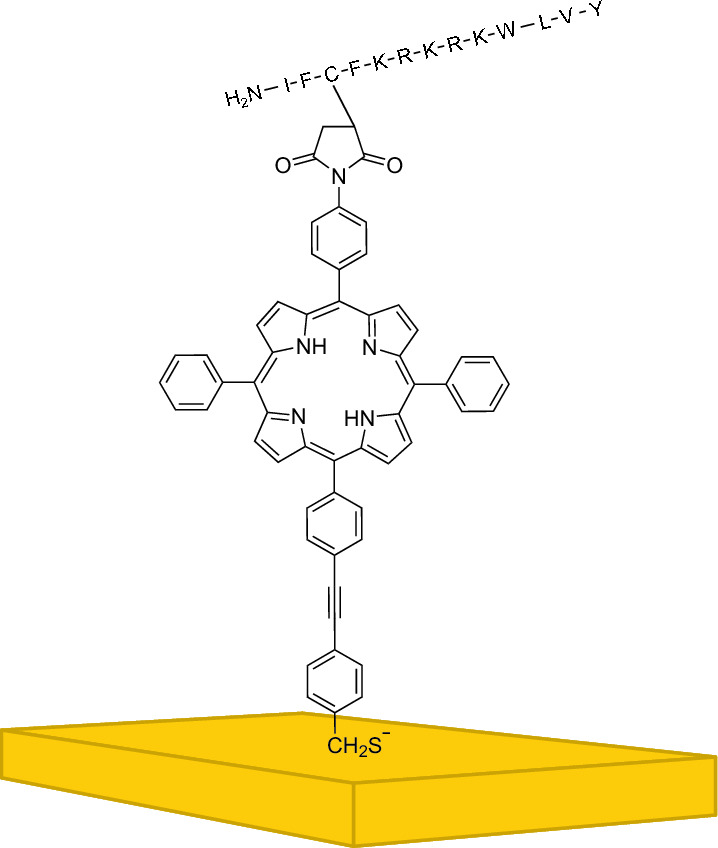
Modified porphyrin for functionalization purposes.

Gold surfaces were functionalized with a modified porphyrin, shown in Fig. 1, by an acid-promoted method, as it is described by Neumann et al.^17,21^.

### Bacteria culture

The handling and cultivation of bacteria were performed under sterile conditions. A single colony of *Escherichia coli (E. coli)* K12 (DSM 498, Leibniz Institute DSMZ—German Collection of Microorganisms and Cell Cultures GmbH, Braunschweig, Germany) was added to 50 mL of liquid lysogeny-broth medium (LB, 10 g/L tryptone, 5 g/L yeast extract, 5 g/L sodium chloride). The suspension was incubated at 37 °C on a shaker incubator (Ecotron, Infors AG, Bottmingen, Switzerland) overnight with a rotation velocity of 150 rpm until an optical density between 0.5 and 0.8 at a wavelength of 600 nm (OD_600_, WPA biowave CO8000, Biochrom Ltd., Cambridge, United Kingdom) was reached. For EGFET measurements, 2 mL of cell suspension were then harvested by centrifugation (Micro Star 17, VWR International, LLC. part of Avantor, Inc., Radnor, PA, USA) with a rotation velocity of 10,000 rpm for 3 min. The cell sediment was washed three times in PBS. The cells were suspended in PBS to a final OD_600_ between 0.6 and 0.8.

### Analytical procedures

**Figure 2:**
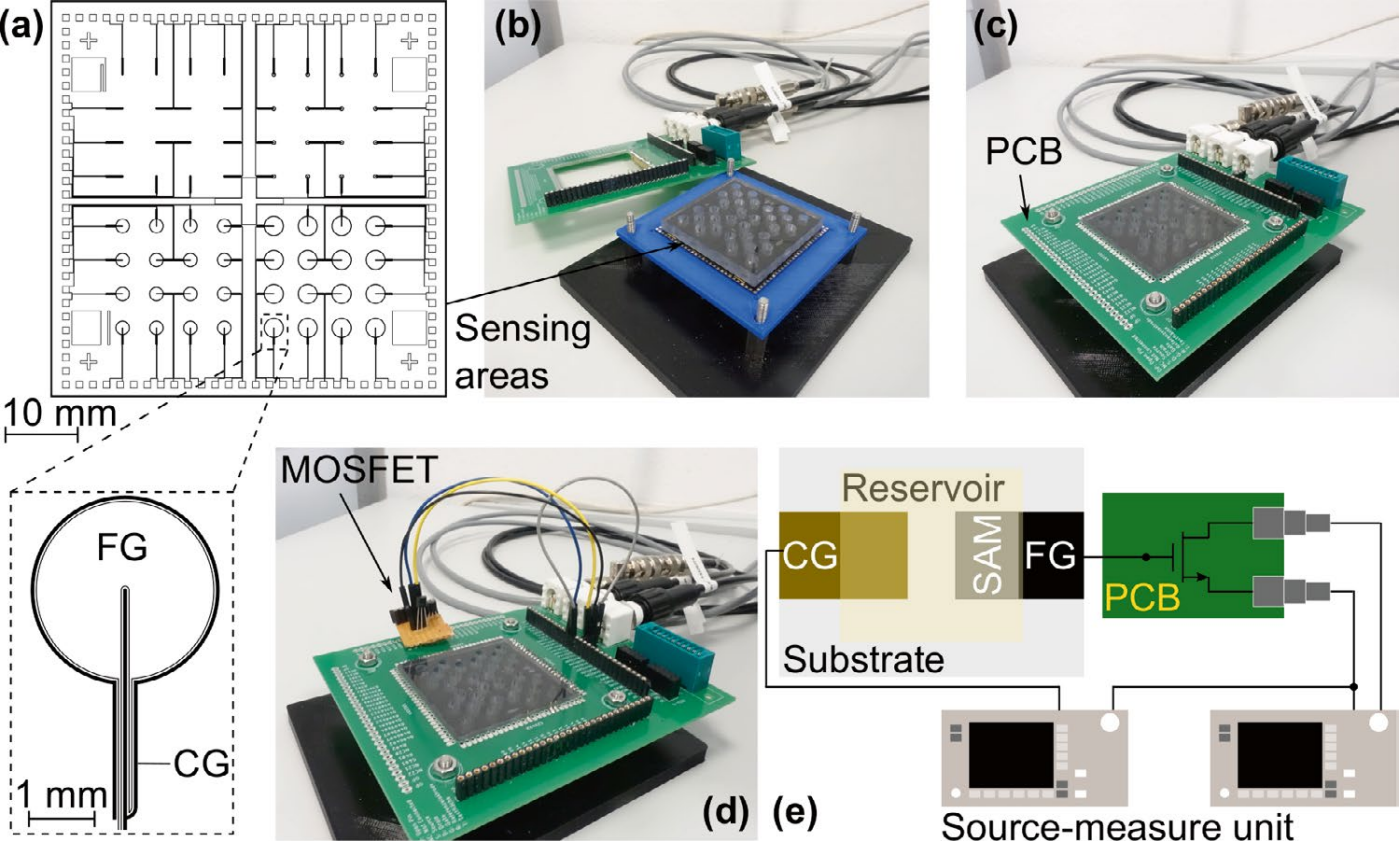
Hybrid setup of the developed EGFET. (**a**) Lithography mask with one sensor area displayed in larger scale underneath. For a better visualization, the area of the control-gate electrode (CG) is filled whereas the floating-gate electrode (FG) is only displayed with a framed line. All closed areas on the lithography mask are filled by a chromium layer. (**b**) Realization of the sensor thin-film substrate. The polycarbonate plate has open holes for the liquid and is stick to the glass substrate. (**c**) PCB for connection purposes between the sensor substrate, the transducer, and the SMUs. (**d**) Connection between a sensor area and the MOSFET by wire jumpers. (**e**) Integration of the SMUs into the measurement setup for recording the transfer characteristics.

**Figure 3:**
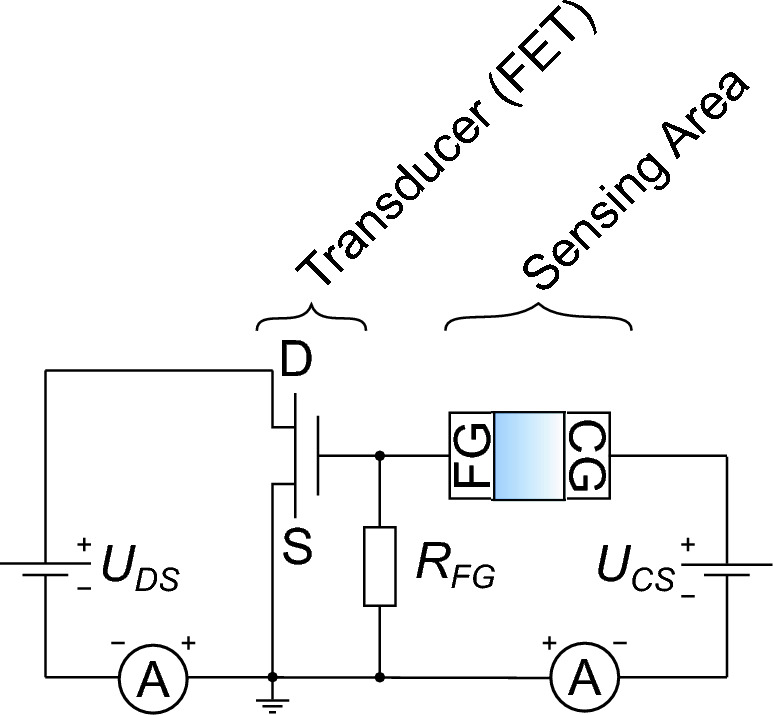
Equivalent circuit of the EGFET with a FET as transducer, the sensing area, and two SMUs including a voltage-source and an amperemeter. Area between FG and CG represents the liquid. D: Drain-electrode, S: Source-electrode, FG: Floating-gate electrode, CG: Control-gate electrode, *R*_*FG*_: Floating-gate resistor, *U*_*DS*_: Drain-source voltage, *U*_*CS*_: Control-gate-source voltage.

The EGFET was realized as hybrid setup. The sensing area was produced by thin-film technology as introduced in the section Sample preparation. A commercial n-channel metal-oxide semiconductor field-effect transistor (MOSFET, LND150N3-G, Microchip Technology Inc., Chandler, AZ, USA) was used as transducer. Figure 2a shows the design of the sensing areas. One of it is displayed in larger scale underneath. The sample features four sections which only differ in the size of the floating-gate electrode (FG). Within one section the sensing areas are identical. A polycarbonate plate with cylindrical holes, as displayed in Fig. 2b, was stuck to the glass substrate to realize the reservoirs which were filled with either the bacteria suspension or the PBS-solution without bacteria. The sensing areas are electrically connected to the developed printed circuit board (PCB) by spring contacts (Fig. 2c). The specific developed PCB enables the electrical connection between the sensing area, the transducer (MOSFET, Fig. 2d), and the source-measure units (SMU, Fig. 2e) to record transfer characteristics. Figure 2 is appropriate subscribed. Figure 3 schematically displays the important details of the measurement setup as equivalent circuit.

Before each measurement, the reservoir was filled either with a bacteria suspension, prepared as explained in the subsection *Bacteria culture*, or with a PBS-solution without bacteria and stored for 10 min at 37 °C. Transfer characteristics were then recorded with a constant drain-source voltage (*U*_*DS*_) with a value of *U*_*DS*_ = 5 V. The voltage applied to the control-gate electrode (*U*_*CS*_) was swept between *U*_*CS*_ = –1 V and *U*_*CS*_ = 1 V in forward and backward manner with a linear potential sweep of 20 mV/s. Each measurement point was integrated over 2 s. The setup was electromagnetically shielded to prevent external noise.

For the second operation mode, a constant voltage of *U*_*DS*_ = 5 V and *U*_*CS*_ = 1 V was first applied to the EGFET for 15 min before the transfer characteristics were measured.

Additional, a transfer characteristic was recorded with only the used transducer (MOSFET) of the EGFET. The voltages applied are the same which were introduced for the EGFET. In contrast, the variable voltage was applied to the gate (*U*_*GS*_) instead of the control-gate electrode. In the following discussions, the transfer characteristics of the EGFET are compared to the results of the MOSFET. Therefore, the variable voltage is described with *U*_*XS*_ on the graphs. X represents *C* for the EGFET and *G* for the MOSFET.

### Theory

**Figure 4:**
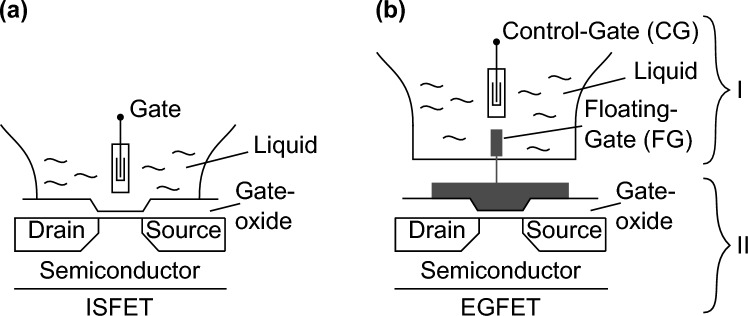
Schematic of an (**a**) ISFET and an (**b**) EGFET physically separated into (I) sensing area and (II) transducer (field-effect transistor) but electrically connected by the floating-gate electrode (FG)^23^.

An EGFET has its seeds in an ion-sensitive field-effect transistor (ISFET)^22^. Figure 4 shows schematically an ISFET as well as an EGFET.

In both transistor configurations, the liquid contains the analyte of interest. For an ISFET, the electrochemical potential at the interface between the liquid and the gate-oxide is depended on the analyte and is further reflected in the measurable current between source and drain^22^. In contrast, the liquid is not in contact with the gate-oxide for an EGFET. The floating-gate electrode (FG) in Fig. 4b is extended to a physically separated sensing area (I)^6,24^. The liquid electrochemically connects the FG with the so-called control-gate electrode (CG) which is comparable with the gate-electrode of the ISFET. The potential of the FG is regulated by the voltage applied to the CG and the electrical characteristic of the liquid. Since the current between source and drain of the transducer (II) is controlled by the potential at the FG, changes are getting measurable equivalent to the ISFET^22^.

## Results and discussion

The following discussion starts to analyze the need of having the floating-gate resistor (*R*_*FG*_) in the measurement setup (Fig. 3). Further, the influence of bacteria on the electrical behavior of the developed EGFET are discussed with results recorded by two operation modes. Finally, the impact of a functionalized sensor area for the detection of bacteria cells are analyzed. The successful functionalization of the sensing area was already shown and discussed in detail by our previous publication with ultraviolet-visible (UV/Vis) spectroscopy, drop-shape analysis (DSA), cyclic voltammetry (CV), and infrared-reflection-absorption spectroscopy (IRRAS). Also the ability of the porphyrin molecule (Fig. 1) to link bacteria cells on the electrode’s surface was verified by fluorescence lifetime imaging microscopy (FLIM)^17^. This work focuses on the detection of bacteria by the developed EGFET.

**Figure 5:**
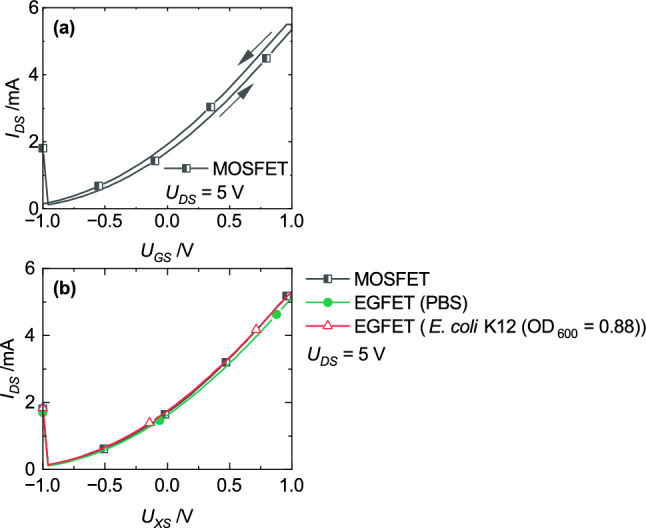
(**a**) Transfer characteristic of the MOSFET without the sensing area (squares) in comparison to (**b**) EGFETs contacted with a PBS-solution (circles) or a bacteria suspension (triangles) on the porphyrin-functionalized sensing area. The measurement results are shown only in forward manner for a clear overview (**b**) due to the almost identical characteristics of the EGFETs compared to the MOSFET results presented in (**a**).

The electrical behavior of the EGFET without *R*_*FG*_ was first analyzed. In the further discussions, the influence of the sensing area on the electrical behavior of the transducer will frequently referred. Therefore, Fig. 5a presents the transfer characteristic of only the used transducer (MOSFET) in the setup of the EGFET. Figure 5b shows transfer characteristics again recorded with the MOSFET but also with two EGFETs. One of them was contacted with a bacteria suspension and the other one with a PBS-solution to clearly relate differences on the attendance of bacteria. Only the results recorded in forward manner are shown to keep a clear overview. The EGFETs showed almost identical characteristics compared to the MOSFET (Fig. 5a) which gives the reason for the reduced presentation of the results.

The reproducibility of the measured behavior was verified with repeating measurements. Three different sensing areas were contacted with a bacteria suspension and another three with a PBS-solution. The characteristics are identical to the results shown in Fig. 5b. Besides reproducibility, the identical characteristics confirm the reliance of the measurement setup. Therefore, all further results were verified by one additional measurement with identical setup.

**Figure 6:**
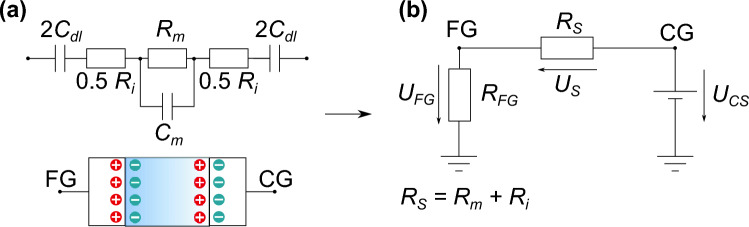
Equivalent circuit of (**a**) two electrodes in contact with a liquid electrolyte^25^ and (**b**) of the sensing area. *C*_*dl*_: Capacitance of the electrochemical-double layer (EDL), *R*_*i*_: Resistor of the EDL, *R*_*m*_: Resistor of the electrolyte, *C*_*m*_: Capacitance of the electrolyte, FG: Floating-gate electrode, CG: Control-gate electrode *R*_*s*_: Simplified resistor for the liquid of the EGFET, *U*_*FG*_: Floating-gate potential, *U*_*CS*_: Control-gate voltage.

The transfer characteristics are almost identical and indicate that the developed EGFET seems not to be sensitive enough to detect differences in the liquid. Only the current between source and drain was measureable but clearly reflects the potential at the FG. Due to the identical transfer characteristics, the potential of the FG (*U*_*FG*_) was only regulated by the applied voltage on the CG but not controlled by the liquid. Based on the published equivalent circuit for two electrodes in contact with a liquid electrolyte by Grossi et al.^25^ (Fig. 6a), the electrical behavior of the sensing area can be translated into a resistor. The capacities are neglectable^25^. The complete equivalent circuit of the sensing area is presented in Fig. 6b.

Based on a voltage divider, Eq. (1) represents *U*_*FG*_ as a function of the liquid (*R*_*S*_), the floating-gate resistor (*R*_*FG*_), and the applied voltage at the CG (*U*_*CS*_).

1 $$U_{{FG}} = \frac{{1}}{{R_{S} /R_{{FG}} + 1}} \cdot {U_{{CS}}}$$

*R*_*FG*_ was infinite during the measurement of the results presented in Fig. 5. Equation (1) simplifies to *U*_*FG*_ ≈ *U*_*CS*_ and confirms the identical transfer characteristics (Fig. 5). In summary, the ideal isolated FG and not the liquid dominates the electrical behavior of the EGFET.

**Figure 7:**
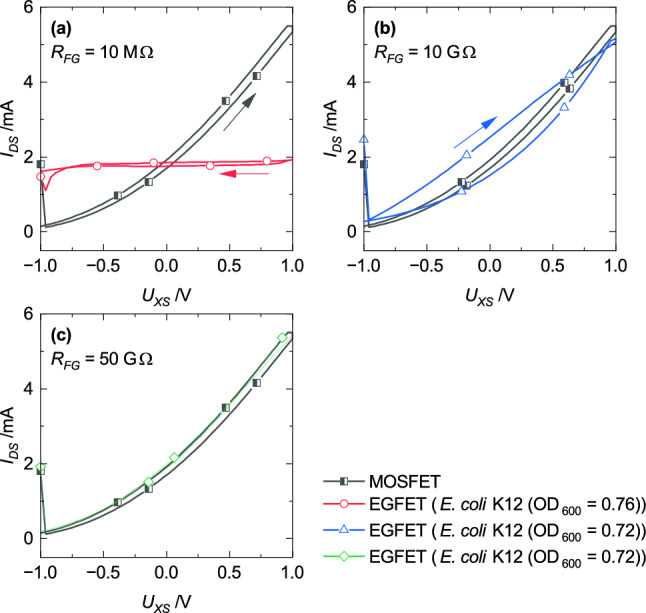
Transfer characteristics of EGFETs with *R*_*FG*_ = 10 MΩ (**a**, circles), *R*_*FG*_ = 10 GΩ (**b**, triangles), and *R*_*FG*_ = 50 GΩ (**c**, diamonds) contacted with a bacteria suspension each in comparison to only the transducer (squares).

According to Eq. (1), *R*_*FG*_ might be necessary to increase the sensitivity towards *R*_*S*_. Resistors with different values were characterized with the setup shown in Fig. 3. Figure 7 shows the appropriate results. (1) The drain-source current measured with *R*_*FG*_ = 10 MΩ as floating-gate resistor is almost constant (*I*_*DS*_ ≈ 2 mA) (Fig. 7a). (2) The regressive transfer characteristic of the EGFET setup with *R*_*FG*_ = 10 GΩ can be separated into a linear and a saturation region. The characteristic shows also hysteresis (Fig. 7b). (3) Fig. 7c presents the measurement result of the setup with *R*_*FG*_ = 50 GΩ which is comparable to the characteristic recorded with the setup that has an ideal isolated FG (Fig. 5).

In detail, the current value detected with measurement setup (1) (*R*_*FG*_ = 10 MΩ) was also measured for *U*_*GS*_ ≈ 0 V with only the MOSFET (Fig. 7a, squares). From this it follows that the transducer in the EGFET setup was also gated with *U*_*FG*_ ≈ 0 V. Hence, *U*_*S*_ is only dominated by the applied voltage (*U*_*CS*_) and not by the liquid. Further, for measurement setup (3) (*R*_*FG*_ = 50 GΩ) the explanation concerning the results recorded with the setup that has an ideal isolated FG is transferable to this result. Finally, the hysteresis recorded with the measurement setup (2) (*R*_*FG*_ = 10 GΩ) can be ascribed to an impact of the liquid on the electrical behavior. Therefore, the measurement setup (2) has achieved the best sensitivity to detect bacteria and was used for further investigations.

**Figure 8:**
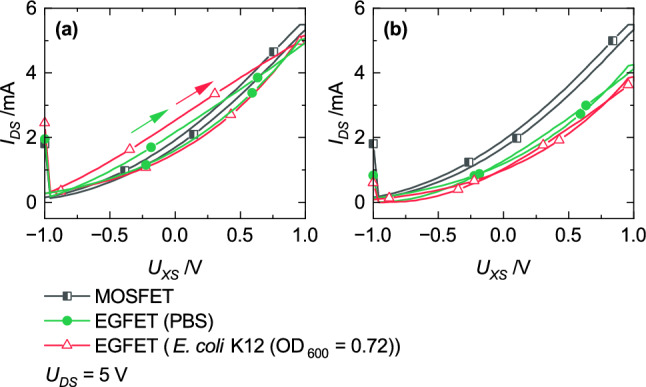
Transfer characteristics recorded (**a**) subsequent after filling the reservoir with either a bacteria suspension or a PBS-solution and (**b**) after application of a constant voltage of *U*_*DS*_ = 5 V and *U*_*CS*_ = 1 V on the EGFET. Circles represent the measurement with a bacteria suspension, triangles with a PBS-solution, and squares represent the transfer characteristic of the MOSFET.

Further analysis for detailed discussions were performed with two operation modes. Both procedures enable the detection of bacteria but different effects dominate the results. The sensing area was prepared according to the measurement procedure explained in the subsection *Analytical procedures*. Transfer characteristics were recorded subsequent after the reservoir was filled with either the bacteria suspension or the PBS-solution (Fig. 8a). In addition, the EGFET was first applied to a constant voltage of *U*_*DS*_ = 5 V and *U*_*CS*_ = 1 V for 15 min and afterwards the transfer characteristic was measured (Fig. 8b).

**Figure 9:**
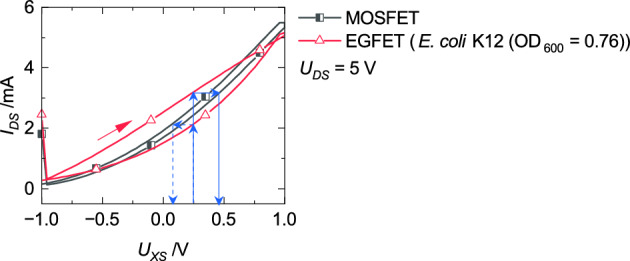
Determination of the floating-gate potential *U*_*FG*_ with a transfer to the MOSFET’s characteristic symbolized by compact arrows for the forward sweep and dashed arrows for the backward sweep.

In the results of the first operation mode (Fig. 8a), *U*_*FG*_ differs according to the forward or backward sweep and results in the measured hysteresis. *U*_*FG*_ can be determined with the help of the MOSFET’s transfer characteristic. Each current value of the EGFET can be transferred to the appropriate characteristic. The corresponding voltage can be determined as *U*_*FG*_. This procedure is shown in Fig. 9.

**Figure 10:**
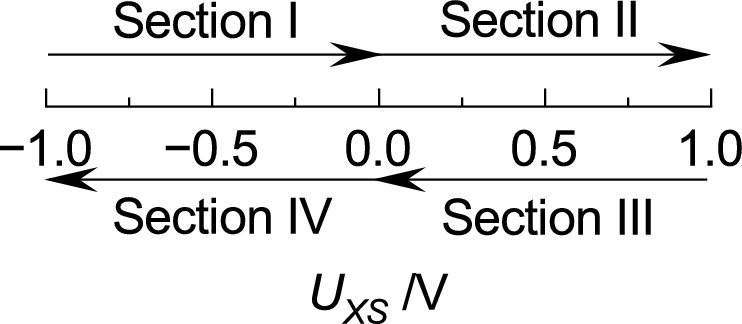
Visualization of Section I, II, III, and IV respectively to Table 1.

For a better visualization of the following discussion, the EGFET’s transfer characteristics are divided into four sections which are listed in Table 1 and visualized in Fig. 10. It is differentiated if the electrochemical-double layer (EDL) at the interface between the electrode and liquid is formed or reduced according to the applied potential (*U*_*CS*_). Section I covers negative *U*_*CS*_ values with an increasing potential sweep. This combination leads to a reduction of the EDL. Section II contains also an increasing potential sweep but positive *U*_*CS*_ values which comes along with an EDL formation. Section III is similar to Section II but with a decreasing potential sweep. This difference leads to a reduction of the EDL. Section IV covers negative *U*_*CS*_ values, equal to Section I, but with a decreasing potential sweep. Therefore, the EDL is formed under this condition.

**Table 1:** Division of the transfer characteristic according to the applied potential and the appropriate EDL.

Section	Applied voltage	EDL
I	*U*_*CS*_ < 0 V; Forward sweep	Reduction
II	*U*_*CS*_ > 0 V; Forward sweep	Formation
III	*U*_*CS*_ > 0 V; Backward sweep	Reduction
IV	*U*_*CS*_ < 0 V; Backward sweep	Formation

As can be seen from Fig. 9, *U*_*FG*_ is higher if the EDL is formed compared to its reduction. Based on the mesh rule for Fig. 6b, a higher *U*_*FG*_ results in a lower *U*_*S*_ at the same operation point. Two different effects can be discussed. Based on Ohm’s law (U = R · I) a change in the resistor or in the current could cause a potential change and even a combination is possible. *R*_*S*_ is invers proportional to the carrier mobility *µ*_*i*_ (Eq. (2) and (3))^26^.

2 $$R=\frac{{1}}{\kappa }\frac{{l}}{A}$$

3 $$\kappa =F\sum_{i}\left|{z}_{i}\right|{\mu }_{i}{C}_{i}$$

$\kappa$ is the conductivity, *l* the distance, *A* the cross-sectional area, *F* the Faraday constant, $\left|{z}_{i}\right|$ magnitude of charge, and *C*_*i*_ the concentration of species *i*^26^.

*U*_*S*_ is smaller during formation of the EDL which is then caused by a higher carrier mobility based on the previous explanation. During reduction of the EDL, the resistance rises. It can be assumed that intermolecular interaction in the EDL might reduce the mobility.

Alternatively or additionally, a change in the flux of charge carriers could cause a change in the potential drop over the liquid. Based on the Nernst-Planck equation (Eq. (4)), the flux of species *i* is a combination of diffusion, migration, and convection^26^.

4 $${J}_{i}\left(x\right)=-{D}_{i}\frac{\partial {C}_{i}\left(x\right)}{\partial x}-\frac{{z}_{i}F}{RT}{D}_{i}{C}_{i}\frac{\partial \phi \left(x\right)}{\partial x}+{C}_{i}\upsilon (x)$$

In addition to the introduced variables, *D*_*i*_ is the diffusion coefficient, $\partial {C}_{i}\left(x\right)/\partial x$ and $\partial \phi \left(x\right)/\partial x$ the concentration and potential gradient, respectively, *R* the gas constant, *T* the temperature, and $\upsilon (x)$ the velocity^26^.

Convection is negligible due to the static setup. At the beginning of the EDL’s formation, the charge carriers are statistically distributed in the liquid. Only migration is the driving force. In contrast, the charges are accumulated on the electrodes during reduction of the EDL. The concentration gradient is at its maximum and causes an impact of the diffusion factor. In summary, the current density is higher for the case of an EDL reduction. A higher flux resulting in a higher potential drop and can also be an explanation for the measured hysteresis.

Higher current values were recorded with the bacteria suspension within the forward scan (Fig. 8a). Thakur et al.^27^ published an increasing electrical conductivity with a higher number of bacteria. Conductivity is proportional to carrier’s mobility (Eq. (3)). Therefore, an increasing conductivity resulting in a lower liquid resistance and further in a lower potential drop over the liquid. In summary, *U*_*FG, Bacteria*_ > *U*_*FG, PBS*_ and confirms the result shown in Fig. 8a.

Further, the transfer characteristics are discussed which were recorded after a constant voltage was applied on the EGFET (Fig. 8b). The absence of hysteresis implies that *R*_*S*_ or *J*_*i*_*(x)* does not have an impact on the result during forward and backward sweep. Nevertheless, the transfer characteristics are shifted towards lower values compared to the measurement of the MOSFET without the sensing area. During the operation, the EDL expands into the bulk in a greater manner when a constant voltage was applied compared to the measurement performed subsequent after filling the reservoir. In Fig. 6, the electrical characteristic of the liquid was simplified transferred into a resistor. Due to the larger expansion of the EDL, the capacitances (*C*_*dl*_) of the equivalent circuit in Fig. 6a cannot further be neglected. The potential drop increases with an increase of the expansion. An increasing potential drop over the liquid results in a lower *U*_*FG*_. The transfer characteristic recorded with bacteria added to the liquid results in even lower current values. It can be assumed that the size of bacteria (2–6 µm) results in an even higher expansion of the EDL and further in a lower *U*_*FG*_ compared to the measurement without bacteria.

**Figure 11:**
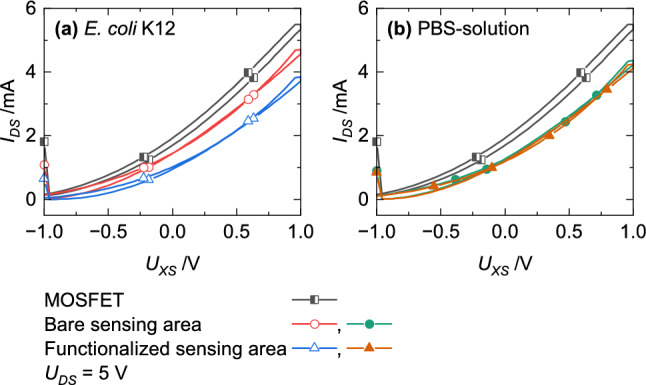
Impact of the functional layer (circles) compared to an EGFET without a functionalized sensor area (triangles) on the transfer characteristic measured with (**a**) *E. coli* K12 and (**b**) PBS-solution.

The last results illustrate that not only the attendance of bacteria but also the functional layer is necessary for its detection (Fig. 11). Only when bacteria have the opportunity to link to the functional layer, a shift to lower current values were recorded (Fig. 11a).

## Conclusion

We introduced an EGFET as biosensor for detection purposes of Gram-negative *E. coli* K12. In the first instance, a hybrid setup was developed. On a large scale, this sensor offers the opportunity that the sensing area can be used as low-cost disposable whereas the MOSFET as transducer can be integrated into the evaluation unit. Alternative setups are in progress wherein the usage of organic instead of the silicon-based FET might lead to a fully integrated thin-film device system to open the opportunity for a lab-on-a-chip sensor and a mass production based on roll-to-roll processing. Two operation procedures were introduced. On the one hand, transfer characteristics were recorded subsequent after filling the reservoir with either the bacteria suspension or PBS-solution. On the other hand, constant voltages were first applied on the EGFET followed by the measurement of transfer characteristics. Changes in the transfer characteristics originate from differences in conductivity and enlargement of the EDL, respectively. Measurements were performed with and without *E. coli* cells. The attendance of bacteria could be clearly verified due to shifts to more negative current values. The results also show that the functionalization of the sensing area is essential. A shift in the transfer characteristic was only recorded if cells were bonded on the porphyrin-SAM.

## References

[1] Fuchs G (2014). Allgemeine Mikrobiologie.

[2] Madigan MT, Martinko JM, Stahl DA, Clark DP (2015). Brock Mikrobiologie kompakt.

[3] Zheng W, Sun W, Simeonov A (2018). Drug repurposing screens and synergetic drug-combinations for infectious diseases. Br. J. Pharmacol..

[4] Zhu L (2016). Development of a double-antibody sandwich ELISA for rapid detection of *Bacillus Cereus* in food. Sci. Rep..

[5] Heo J, Hua SZ (2009). An overview of recent strategies in pathogen sensing. Sensors.

[6] Gutiérrez-Sanz Ó, Andoy NM, Filipiak MS, Haustein N, Tarasov A (2017). Direct, label-free, and rapid transistor-based immunodetection in whole serum. ACS Sens..

[7] Zhang A, Lieber CM (2016). Nano-bioelectronics. Chem. Rev..

[8] Torsi L, Magliulo M, Manoli K, Palazzo G (2013). Organic field-effect transistor sensors: a tutorial review. Chem. Soc. Rev..

[9] Sheibani S (2021). Extended gate field-effect-transistor for sensing cortisol stress hormone. Commun. Mater..

[10] White SP, Dorfman KD, Frisbie CD (2015). Label-free DNA sensing platform with low-voltage electrolyte-gated transistors. Anal. Chem..

[11] White SP, Frisbie CD, Dorfman KD (2018). Detection and sourcing of gluten in grain with multiple floating-gate transistor biosensors. ACS Sens..

[12] White SP, Sreevatsan S, Frisbie CD, Dorfman KD (2016). Rapid, selective, label-free aptameric capture and detection of ricin in potable liquids using a printed floating gate transistor. ACS Sens..

[13] Palit S (2020). Ultrasensitive dopamine detection of indium-zinc oxide on PET flexible based extended-gate field-effect transistor. Sens. Actuat. B Chem..

[14] Ligler FS (2007). The array biosensor: portable, automated system. Anal. Sci..

[15] Pujol-Vila F, Villa R, Alvarez M (2020). Nanomechanical sensors as a tool for bacteria detection and antibiotic susceptibility. Front. Mech. Eng..

[16] Karbelkar AA, Furst AL (2020). Electrochemical diagnostics for bacterial infectious diseases. ACS Infect. Dis..

[17] Neumann L (2021). A2BC-type porphyrin SAM on gold surfaces for bacteria detection applications: synthesis and surface functionalization. Materials.

[18] Ishida M, Naruta Y, Tani F (2010). A porphyrin-related macrocycle with an embedded 1,10-phenanthroline moiety: fluorescent magnesium(II) ion sensor. Angew. Chem. Int. Ed..

[19] Bettini S, Pagano R, Borovkov V, Giancane G, Valli L (2019). The role of the central metal ion of ethane-bridged bis-porphyrins in histidine sensing. J. Colloid Interface Sci..

[20] Vaishnavi E, Renganathan R (2014). “Turn-On-Off-On” fluorescence switching of quantum dots-cationic porphyrin nanohybrid: a sensor for DNA. Analyst.

[21] Sathyapalan A (2005). Preparation, characterization, and electrical properties of a self-assembled meso-pyridyl porphyrin monolayer on gold surfaces. Aust. J. Chem..

[22] Guliga, H., Abdullah, W. F. H. & Herman, S. H. Extended gate field effect transistor (EGFET) integrated readout interfacing circuit for pH sensing. In: *Proceedings of the 2nd International Conference on Electrical, Electronics and System Engineering (ICEESE)*, Kuala Lumpur, Malaysia (2014).

[23] Bergveld P (2003). Thirty years of ISFETOLOGY: What happened in the past 30 years and what may happen in the next 30 years. Sens. Actuat. B Chem..

[24] White SP, Dorfman KD, Frisbie CD (2015). Operating and sensing mechanism of electrolyte-gated transistors with floating gates: building a platform for amplified biodetection. J. Phys. Chem. C.

[25] Grossi M, Riccò B (2017). Electrical impedance spectroscopy (EIS) for biological analysis and food characterization: a review. J. Sens. Sens. Syst..

[26] Bard AJ, Faulkner LR (1980). Electrochemical methods: fundamentals and applications.

[27] Thakur B (2018). Rapid detection of single *E. coli* bacteria using a graphene-based field-effect transistor device. Biosens. Bioelect..

