# Author Correction: Functionalization of an extended-gate field-effect transistor (EGFET) for bacteria detection

**DOI:** 10.1038/s41598-023-46380-w

**Published:** 2023-11-14

**Authors:** Lea Könemund, Laurie Neumann, Felix Hirschberg, Rebekka Biedendieck, Dieter Jahn, Hans-Hermann Johannes, Wolfgang Kowalsky

**Affiliations:** 1 Institut für Hochfrequenztechnik, Technische Universität Braunschweig, 38106 Braunschweig, Germany; 2 Institute of Microbiology and Braunschweig Integrated Centre of Systems Biology (BRICS), Technische Universität Braunschweig, 38106 Braunschweig, Germany; 3 Cluster of Excellence PhoenixD (Photonics, Optics, and Engineering—Innovation Across Disciplines), 30167 Hannover, Germany

Correction to: *Scientific Reports* 10.1038/s41598-022-08272-3, published online 15 March 2022

The original version of this Article contained an error in Figure 11 where the legends were interchanged. The graphs which belonged to the “bare sensing area” was described as “functionalized sensing area” and vice versa.

**Figure 11:**
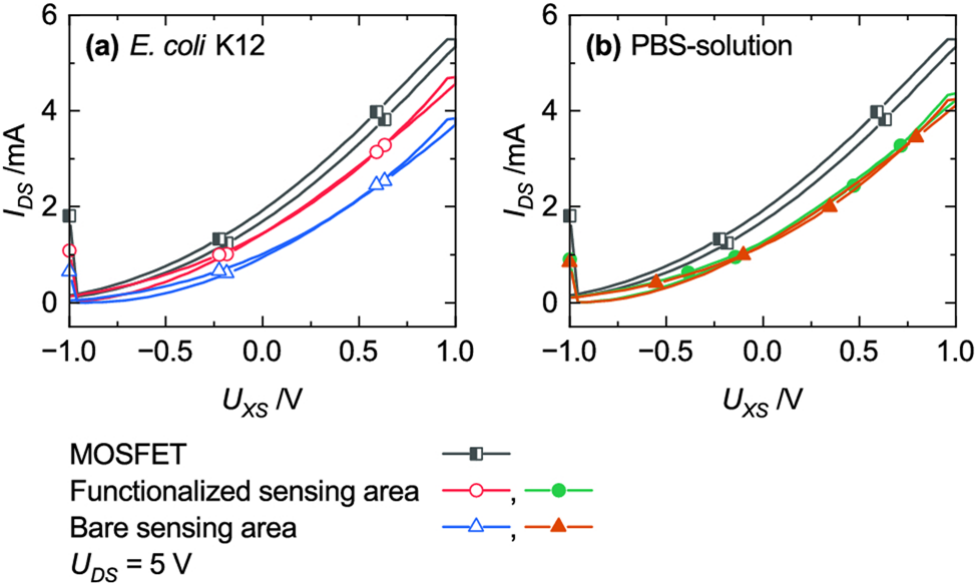
Impact of the functional layer (circles) compared to an EGFET without a functionalized sensor area (triangles) on the transfer characteristic measured with (**a**) *E. coli* K12 and (**b**) PBS-solution.

The original Figure 11 and accompanying legend appear below.

The original Article has been corrected.

